# Development and Validation of LiDAR Sensor Simulators Based on Parallel Raycasting

**DOI:** 10.3390/s20247186

**Published:** 2020-12-15

**Authors:** Guilherme Ferreira Gusmão, Carlos Roberto Hall Barbosa, Alberto Barbosa Raposo

**Affiliations:** 1 Postgraduate Programme in Metrology, Pontifical Catholic University of Rio de Janeiro, Rua Marquês de São Vicente, 225, Gávea, Rio de Janeiro 22451-900, Brazil; gusmaof@tecgraf.puc-rio.br; 2 Tecgraf Institute, Pontifical Catholic University of Rio de Janeiro, Rua Marquês de São Vicente, 225, Gávea, Rio de Janeiro 22451-900, Brazil; abraposo@tecgraf.puc-rio.br

**Keywords:** sensor simulator, LiDAR, synthetic point cloud, remote sensing, raycasting

## Abstract

Three-dimensional (3D) imaging technologies have been increasingly explored in academia and the industrial sector, especially the ones yielding point clouds. However, obtaining these data can still be expensive and time-consuming, reducing the efficiency of procedures dependent on large datasets, such as the generation of data for machine learning training, forest canopy calculation, and subsea survey. A trending solution is developing simulators for imaging systems, performing the virtual scanning of the digital world, and generating synthetic point clouds from the targets. This work presents a guideline for the development of modular Light Detection and Ranging (LiDAR) system simulators based on parallel raycasting algorithms, with its sensor modeled by metrological parameters and error models. A procedure for calibrating the sensor is also presented, based on comparing with the measurements made by a commercial LiDAR sensor. The sensor simulator developed as a case study resulted in a robust generation of synthetic point clouds in different scenarios, enabling the creation of datasets for use in concept tests, combining real and virtual data, among other applications.

## 1. Introduction

3D imaging technology is capable, through the use of sensors and specialized cameras that can be coupled in autonomous and remotely operated vehicles, of collecting data from a target object or region and processing them on the computer to generate precise metric data, with a resolution capable of reaching the order of millimeters [1,2,3]. One form of displaying these datasets is in sets of points in the same coordinate system with geospatial/metric information, called point clouds.

Among the 3D imaging systems, the Light Detection and Ranging (LiDAR) device, a light-based active optical 3D imaging sensor, has been receiving more and more attention, both from the industrial sector and the academic environment. The accuracy and speed of data acquisition, coupled with the ability to embed it in unmanned or remotely operated vehicles, have enabled a range of interesting applications such as autonomous cars performing real-time mapping of their surroundings using LiDAR sensors with machine learning networks helping with environment interpretation [4,5]. Extensions of forests can have their canopies examined using point clouds generated by LiDAR systems embedded in drones or airplanes [6,7,8]. Similarly, the use of such technology in archeology has been helping to identify the ruins of cities long eroded by time [9,10,11]. Submarine operations already use LiDAR for inspections of oil wells and shipwrecks [12,13,14].

Although many applications using 3D imaging sensors, like LiDAR, are successful [3,4,6,12,13,14], some issues are associated with the technology:

- Raw data gathering is directly dependent on how many sensors are used and how large is the scanned target. So, the bigger the target is, the slower and more expensive the operation will be [4,10,15];
- There are situations impossible to be tested due to safety restrictions and preservation of human welfare, as in data acquisition for the training of autonomous vehicles in cases of a car crash and pedestrian or animal run over [4,16];
- Scannings done in hostile environments have a higher risk of equipment damage, like in underwater surveys of drowned wreckage sites [13,14,17];
- The technology hardware and equipment are still expensive at the time of this publication [5,14]; and
- Due to the gigantic amount of points typically present in point clouds, semantic segmentation (data annotation with classification labels for each point or pixel) becomes onerous and time-consuming [4,18,19,20].

These aspects hinder applications that need an enormous amount of diverse data, like creating trainable datasets for deep learning. This limitation is known as the data-hungry effect [4,15,19,20,21]. Many are the efforts to bypass or solve this barrier, as seen in the literature review paper by Gao et al. [20]. One trending solution is the development of sensor simulators, also known as virtual scanning. The technique consists of the sensor simulator interacting with a 3D virtual scenario, yielding synthetic data with fully or semi-automatic annotation [19,20,21].

Since, in a simulation, the environment and situations can be controlled, it is possible to reproduce many scenarios and experiments, even those with potential risks for equipment or personnel. Consequently, these procedures generate more point clouds datasets in a shorter time, depending only on the computational power available [4,6,19,20]. Some of the limitations present on data acquisition on the field can be observed in [21], in which the entire scanning process took five days with a car coupled with imaging sensors, surveying the target zone only during daylight hours. These simulators can also be useful in education, allowing data to be generated in several different laboratory situations, allowing students to better understand imaging technologies before using real devices [16].

In the case of LiDAR simulators, most of the related work is in the sphere of autonomous vehicles. Hanke et al. [4] produced a sensor simulator based on LiDAR, using the NVIDIA raytracing engine Optix [22], adapted inside the Vires’ VTD driving simulator, producing datasets for training autonomous cars. The article also shows some guidelines for sensor simulator development, classifying it into two types: “sensor measurement model” and “sensor error model”. A similar method was developed by Wang et al. [18], where synthetic point clouds with semantic labels of detected objects were generated with a raycasting LiDAR simulator using CARLA [18] autonomous driving simulator, and later experiments show the effectiveness of mixed datasets for the deep learning method training. Fang et al. [23], unlike previous simulators that entirely rely on Computer Graphics models and game engines, proposed a novel LiDAR simulator that augments real point clouds with synthetic obstacles (e.g., vehicles, pedestrians, and other movable objects). Manivasagam et al. [24] built a large dataset of 3D static maps and 3D dynamic objects by scanning several cities with LiDAR. They argue that by leveraging real data, they can simulate the complex world more realistically compared to employing virtual worlds built from CAD models. They also implemented a neural network to produce deviations that cause ray drops. Zhao et al. [25] proposed a simulation-to-real domain adaptation (SRDA) to train a deep neural network (DNN) using unlimited synthetic data with automatically generated labels. The simulator consists of self-supervised dropout noise rendering, statistics-invariant and spatially adaptive feature alignment, and transferable segmentation learning. Hadj-Bachir and de Souza [26] presented a LiDAR simulator with laser beam propagation and energy attenuation in clear weather, fog, rain, and other harsh conditions.

Beyond autonomous vehicle training, the most prominent works are in robotics and forestry. Tallavajhula [15] shows in his thesis a LiDAR simulator for robotic navigation and justifies the development of these simulators as an aid in the development and improvement of robot navigation. Boucher et al. [27] simulate GEDI (NASA Global Ecosystem Dynamics Investigation) spaceborne LiDAR waveforms using airborne LiDAR datasets to analyze the mortality rate of hemlock plots by an insect infestation of hemlock wooly adelgid. Yun et al. [28] developed a new multiple-scan methodology to evaluate how much occlusion affects the computation of tree leaf area using simulated LiDAR point clouds from different trees 3D models.

The sensor simulator presented in this manuscript comes from a subsea survey context, mainly in the oil and gas field. As the depths of the ocean are a very hostile environment, plus the expensive equipment, it is impossible to acquire an adequate amount of data for applications such as creating datasets to train deep learning systems [12,15,19]. Thus, generating synthetic point clouds would help to create more data for proof of concept, better planning of data acquisition on-field, and data augmentation in the subsea area [19].

During the period of this research, it was evident that the sensor simulators in the current literature were built from the perspective of the target problem, and this forces new research projects to develop the simulator from scratch [4,6,15,16,18,23,24,25,26,27,28]. So, the main collaborations of the present work to the field are:

- A guideline for LiDAR simulator development for generating synthetic dataset with semiautomatic annotation to easily adapt for different fields of research;
- The development of a sensor simulator based on both sensor models mentioned in [4], therefore called in this paper as sensor hybrid model [29]; and
- A method for calibration of the sensor simulator by comparison with a real sensor.

This manuscript is structured as follows: Section 2 presents the LiDAR sensor overview and the methods used to develop the sensor simulator named optScan; the validation experiment and the sensor simulator results are given in Section 3; conclusions of the manuscript, including recommendations for future works, are described in Section 4.

## 2. Materials and Methods

### 2.1. LiDAR

**Figure 1:**
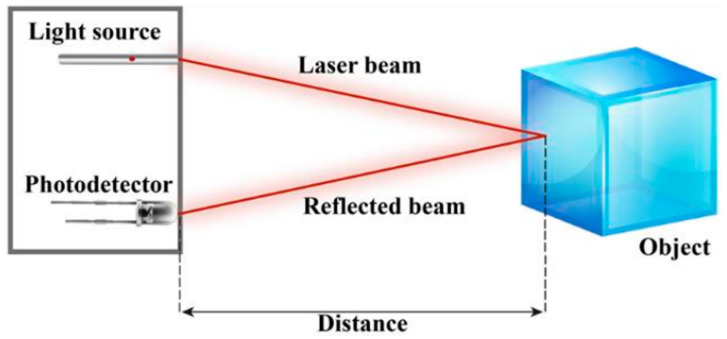
LiDAR ToF technique: The sensor shoots a laser beam from its light source and triggers a time counter, so, when the photodetector receives the returning signal, the system registers the total time of the laser flight [29].

As highlighted in the first section of this paper, the sensor of interest is the LiDAR, a 3D imaging system able to gather distance measurements based, usually, on time of flight (ToF) techniques [1,2,3]. These techniques use the time it takes from a signal to leave and return to the sensor system and calculate the distance traveled by the signal until reaching a reflective surface, as seen in Figure 1 [29]. The scheme shows the sensor’s ToF technique.

The distance can be found with the following equation in most typical LiDAR applications:

(1) $$d=\frac{c\cdot t}{2},$$

where *d* is traveled distance, *t* is the measured time of flight, and *c* is the speed of light [1,2,3].

The raw data gathered is then used in geometric algorithms to calculate the tridimensional spatial points of the scanned surface, which compose the point cloud. Each point in this type of dataset is placed following its 3D position vector (x, y, z). Beyond this, they can also contain information about geopositioning, elevation, normal vector, semantic labels, and many others [1,2,3,9,10,12]. The precision of the LiDAR systems with the richness of information contained in point clouds is why this technology is so widespread in such diverse areas of knowledge.

### 2.2. Sensor Simulators

Most of the presently available 3D imaging simulators are developed by the following guideline: develop or find a suitable virtual scene builder; model a sensor behavior algorithm that will perform the 3D imaging simulation; and, finally, validate the model-generated synthetic data against real data [4,6,15,18,19].

The virtual scene is a digital representation of the scanning target, be it a large outdoor area or a single object. The scene is built using 3D model files that contain information about an object in three dimensions, including position coordinates and many other characteristics. They can be CAD type models, objects used in games, or even models created from point clouds [4,6,15,18,19].

The primary device in a 3D imaging system is its imaging sensor, as it will dictate which data acquisition methods can be used [1,2,3]. The imaging sensor’s digital counterpart is the sensor behavior model, a mathematical model based upon the behavior of a designated sensor and its techniques for data acquisition [4,6,15,18,19]. In the case of 3D imaging methods for generating point clouds, this translates into how to emulate the acquisition of raw data by a real sensor, whether from passive or active 3D optical imaging methods, in the form of images or distances [1,2,4,6,15]. The crucial factor for a robust point cloud generation is the quality of the modeled behavior in the sensor simulator since the real raw data are also processed by computational algorithms to create the target’s point clouds [1,2,4,6,15].

**Figure 2:**
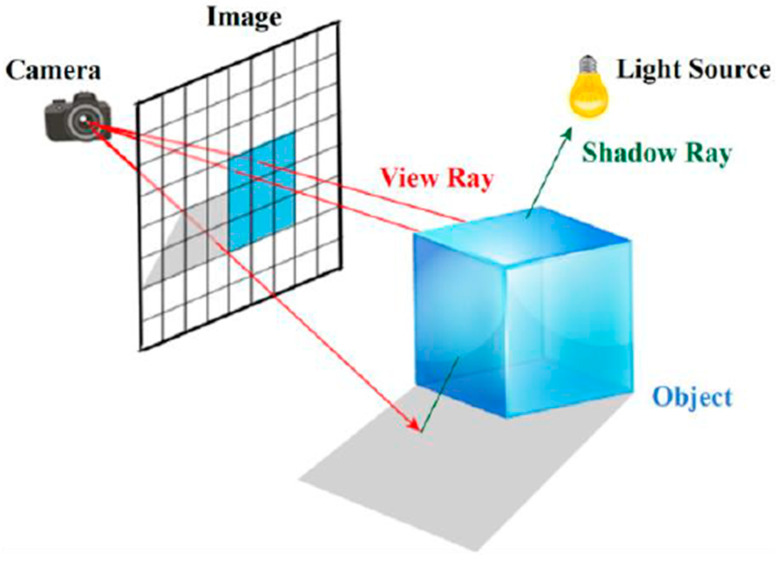
Raytracing’ principle [29].

For LiDAR-based models, it is necessary to replicate the time of flight (ToF) technique, which is the LiDAR operating principle. The literature is leaning towards the use of raytracing, a computer graphics family of algorithms for image rendering, in some form to model the ToF behavior [4,6,15,18,19]. The algorithm traces, for each pixel on the screen, the path of a ray, from the projection center (eye/camera), passing through the pixel, until there is a collision of this ray with an object in the virtual scene, as illustrated in Figure 2. Given this collision, the algorithm returns the color and distance of the object hit, ending the raycasting step. The other possibility is to explore the raytracing algorithm recursiveness. This allows the creation of new rays for multiple reflections, based upon the object surface properties and illumination model [22].

The LiDAR simulators can be easily divided into two categories, presented in Hanke et al. [4], as follows:

- “Sensor measurement model”: models based upon a real sensor measurement technique and the sensor orientation in the scene; and
- “Sensor error model”: models based upon the statistical errors perceived in the measurements made by a real sensor, considering systematic and random errors.

One example of such a simulator is shown in the article by Xiangyu et al. [30]. Here a LiDAR simulator built in a virtual scene can generate a point cloud for each defined scanning position, resulting in a synthetic point cloud.

The last stage of a sensor simulator development is the validation of the behavior model, guaranteeing the robustness of the synthetic point clouds generation. The literature shows two manners for validation:

- By analyzing the error between an on-field point cloud and a synthetic point cloud from the on-field target’s digital counterpart with similar scanning parameters of the real scanning [4,6,15,18]; or
- Through the analysis of the primary signal generated by the sensor simulator, using a real sensor as a reference, with both measurements made of a target and its digital counterpart with similar scanning parameters. This method is a form of calibration for the sensor simulator [4,30].

### 2.3. optScan: A Modular LiDAR Sensor Simulator

As mentioned in the first section, this research aimed to develop a guideline for LiDAR simulator development for generating synthetic datasets with semiautomatic annotation, so it becomes easier to adapt the simulator to different fields of research. To achieve this goal, we propose a modular approach that views the simulator components as autonomous blocks, facilitating the replacement or implementation of its components.

**Figure 3:**
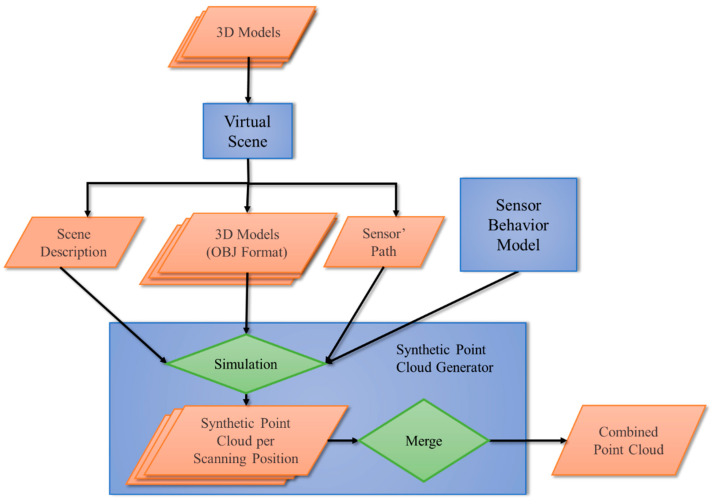
optScan structure flowchart: Parallelograms are inputs, rectangles are modules, and diamonds are internal applications essential for generating Synthetic Point Clouds.

Following this idea, the sensor simulator, dubbed optScan [29], was built with the block structures shown in the flowchart of Figure 3. The rectangles represent the components modules that build the sensor simulator. The parallelograms represent the input and/or outputs of the modules. The diamonds are internal procedures essential to the Synthetic Point Cloud Generator module.

The optScan simulator [29] has three fundamentals modules: Virtual Scene, Sensor Behavior Model, and Synthetic Point Cloud Generator. Each module contributes to different aspects of the LiDAR system scanning process that will result in synthetic point clouds at the end of the pipeline.

#### 2.3.1. Virtual Scene

It is necessary to have a platform where the user can build a scene based on the desired target. As seen earlier in this paper, most of these platforms are long-established simulators, especially in the automobilistic area, wherein the sensor simulator is created [4,18,23,24,25,26,27,28]. Here we propose a different approach: to develop or adapt a virtual scene builder so the user can build a scene and choose the sensor scanning locations. This module should output files with scene characteristics and a sensor scanning path that can be used in other modules to generate the raw data and synthetic point clouds.

To achieve this goal in the optScan simulator, the Virtual Scene module was developed with the Unity 3D engine [31]. The engine offers numerous tools to work with 3D and 2D applications, and it is also free under US$ 100,000.00 earnings. These tools allow the user to easily assemble a virtual scene with 3D objects. Each of these objects has important properties that will be imported to the other modules, like position, rotation, and scale in the virtual world. Another important property of the object is the ID, a unique identification number for each class of object present in the current scene. The user classifies this object when creating its Prefab, a template with defined properties and configuration components that works as a reusable asset [31]. As the ID property will be passed on to the point cloud automatically in the Synthetic Point Cloud Generator module, assigning class labels to objects turns the process semi-automatic. Although semi-automatic annotation will need the user to label an object at least once, it will be harder to have semantic misclassifications in the point clouds [18,19,20].

**Figure 4:**
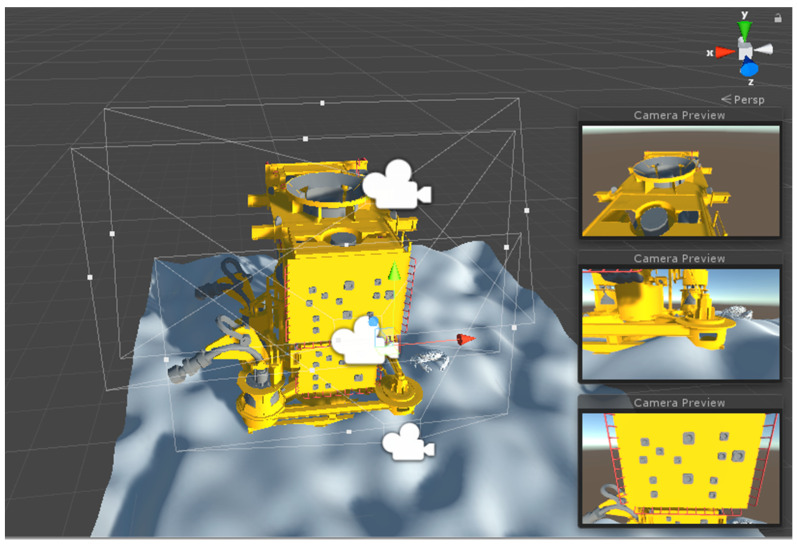
Definition of the sensor path for scanning an oil well Christmas tree. The cinecameras are Unity camera objects used to represent the sensor scan position. The camera preview screens show what the device is seeing inside its field of view [29].

After the scene is built, the user can use the camera object in Unity, a device that displays the world to the player when running the project on game mode [31], to choose the positions that the sensor simulator will scan later in the other modules. The scanning is considered with a fixed position, so the sensor doesn’t move while gathering data. Figure 4 illustrates this process, where the user creates cameras at different positions to define the scanning position and orientation of a target, in this case, an oil well Christmas tree. This feature also helps to see if the synthetic point clouds generated have coherence with the expected view seen in Unity’s camera preview.

Finally, the module should process the virtual scene and output files containing the description of each object’s properties in the scene and the position and orientation of each defined camera. In the case of the optScan simulator, three files are generated:

- Scene Description: Contains the object position, scale, rotation, and ID properties mentioned earlier for each object of the virtual scene and a few other commands for the other modules to interpret the virtual scene for scanning;
- OBJ 3D models: In the case of the optScan, one of the modules needs 3D files with the extension .obj. So, the module converts the Prefab object to .obj files; and
- Sensor Path: Contains the position and orientation of each camera defined as a sensor scanning position by the user.

#### 2.3.2. Sensor Behavior Model

The sensor behavior model is the heart of any sensor simulator, as it is how the simulator will gather data from the digital targets [4,6,15,18,23,24,25,26,27,28,29]. This research sought to create a hybrid model, i.e., joining a model based on measurements [4] with a model based on errors [4] as an attempt to increase accuracy on the raw data acquisition of the virtual scanning in comparison with ground truth (here a real LiDAR sensor) [29].

For the measurement model, it is necessary to replicate the time of flight (ToF) technique, which is the LiDAR operating principle. As seen earlier in this paper, the most used technique is adapting raytracing algorithms, especially raycasting, where the ray path is traced between the source and the object surface hit [18,23,24,25,26,27,28,29]. One good engine to develop the sensor simulator [4,30] is Nvidia’s OptiX raytracing engine [22], as the API allows using a huge set of raytracing tools and algorithms with a high degree of parallelism, that is, the processing of each ray can be performed independently, reducing the total time of computational calculations [22].

Some functions and characteristics of the raycasting algorithm should be adapted to better model the simulator sensor, so the desired conditions and metrological parameters match a real LiDAR sensor characteristic. Below, we propose some adaptations:

- Spherical Plane: LiDAR sensors usually scan an area within an arc of circumference, with a pitch angle defined by the manufacturer. Hence, the LiDAR scanning follows a spherical plane. As the raytracing algorithms use the cartesian plane to render images, the spherical coordinates must be converted into rectangular coordinates. The following trigonometric equations do such conversion:
  (2) x=r·cos(θ)·sin(φ) ,
  (3) y=r·sin(θ)·sin(φ), and
  (4) z=r·cos(θ),
  where *r* is the radius of an arbitrary sphere based upon the sensor scanning direction vector, θ is the yaw angle, and φ is the pitch angle. This brings about conical distortions typical of scanning with LiDAR, as the shooting rays will follow coordinates according to the yaw and pitch angles [4,18,30].
- Shooting angle and scanning area: The LiDAR sensor shoots its laser beam with an angular step within the scanning area. Thus, the same rule must be applied to the sensor behavior model. As each ray during raytracing will transverse a pixel, the total covered area will be equal to the screen size. So, we need to limit this area within the LiDAR scanning angle. By using the rule of three between the current pixel and total scanning area (yaw for azimuth angle or pitch for elevation angle), the following equations were obtained:
  (5) $$\theta =\frac{pixel}{width}\cdot \theta_{max}-\frac{\theta_{max}}{2},\;\mathrm{and}$$
  (6) $$\varphi =\frac{pixel}{height}\cdot \varphi_{max}-\frac{\varphi_{max}}{2},$$
  where *pixel* is the current pixel being traversed by a ray, *width* is the sensor simulator screen width, *height* is the sensor simulator screen width, $\theta_{max}$ is the maximum yaw, and $\varphi_{max}$ is the maximum pitch. When subtracting half the maximum angle, the sensor behavior model will have a central angle at zero and its angles will belong to the interval [−maximum angle/2,+maximum angle/2], as is the default in LiDAR devices [1,2,3,5].
- Maximum detection range: The maximum distance a ray can hit an object should be limited according to the operating distance of a LiDAR. This means that objects outside this limit are not registered by the sensor behavior model, as occurs with a real sensor [1,2,3,5].
- Raycasting: As shown in the literature, most researchers don’t use rays’ reflections, as they depend on the object’s materials and complex phenomena, like light scattering [20,21,22,23,24,25,26,27,28]. Only the raycasting step, tracing a path between the ray and an object collision, is sufficient to produce synthetic point clouds [20,21,22,23,24,25,26,27,28]. Nevertheless, using the full power of raytracing could bring more realism to virtual scanning, especially in experiments embedded in turbid environments, like rain and the sea [4,15,26].

For the error model, there is a focus on modeling the measurements standard deviations caused by the optical properties and conditions of the environment and those coming from the sensing device. Here we propose an empirical approach through experimental tests with a real LiDAR sensor, focusing only on the errors coming from the device:

1. The researcher should choose a LiDAR(s) that fits their work. It will be the cornerstone of the error model;
2. A target with simple geometry needs to be chosen (like a cube, cylinder, etc.) and with low reflectivity, to better isolate the errors coming from the environment;
3. The LiDAR should be placed at a fixed height with its center aimed at the target’s middle section;
4. Measure the distance between the sensor and target with a calibrated measurement device;
5. During the scanning, choose the yaw and pitch scanning area;
6. Scan the target as many times as possible, taking care that the sensor stays put and nothing covers or crosses the zone between sensor and target;
7. The sensor will generate a large number of points. Filter those with null distance;
8. Find the statistical properties of this data (standard deviations, mean, etc.);
9. These statistical properties can be used to obtain an equation that gives the error for each using curve fitting; and
10. Finally, add Gaussian noise to the model to represent the random errors in the measurement.

As a case study, the optScan error model was built with this method. The chosen device was the LiDAR URG-04LX-UG01 from Hokuyo [32], which has the following characteristics:

- Infrared laser with wavelength *λ* = 785 nm.
- Yaw scanning area: 240° (angular resolution ~ 0.3516°)|No pitch scanning area.
- Two operating ranges: 1st range, 60–1000 mm, with a measurement uncertainty of 30 mm. 2nd range, 1000–4000 mm with a measurement uncertainty of 3% of the detected distance.

To better isolate the device’s errors from those coming from the environment, the experiment scanned a target with a simple geometry of cylindrical shape and low reflexivity material. The sensor was centralized with the cylindrical object at middle height. The process was carried out sixteen times for distances ranging from 500 mm to 2000 mm between the central beam of the sensor and the middle of the target, to cover most of the sensor’s detection range (60–4000 mm), resulting in 8000 scans. A Bosch laser measuring tape, with a measurement uncertainty of 1.5 mm, was used as a reference to ensure the correct positioning of the device. The points with null distance were removed. Then, the statistical properties of this set were used to obtain an equation that yields the error in mm for each distance *d* with curve fitting, shown below. The RMSE was 3.69 mm.

(7) $$error\left(d\right)=-5.139\cdot 10^{6}\cdot d^{2}+9.92\cdot 10^{-4}\cdot d+15.66,$$

The systematic error of 15.66 mm can be explained by a mismatch in the respective origins of the LiDAR and of the measuring tape. Additionally, a Gaussian noise *N*(0,3) was added to represent the random errors in the measurements, which were estimated based on the residuals of the quadratic curve fitting.

#### 2.3.3. Synthetic Point Cloud Generator

Finally, this is the module responsible for generating synthetic point clouds. It comprises two main steps: simulating the virtual scanning and creating a single 3D synthetic point cloud from all the data generated. These two steps could also be separated into two new modules if the researcher prefers to maintain both applications independently.

The simulation step should recreate the digital targets mathematically within a raytracing engine or similar tools, like the OptiX engine, through the files created in the Virtual Scene module. Then, the virtual scanning is performed by the sensor behavior model, which obtains the intersection distance when a ray collides with an object. In the case of the OptiX engine, the intersection distance is found by ray-geometry intersection tests. Each triangle from the 3D object mesh in the scene is included in a logical tree structure. While traversing this tree, the algorithm initially filters the triangles for candidates that can be touched by the ray, thus reducing the computational load. For the remaining triangles, the algorithm then applies well-known ray-triangle intersection formulas, and computes distances, normals, texture coordinates, and other attributes based on the collided position [22]. Given these distances, the 3D position can be calculated, thus creating the point relative to the surface collided by the ray. This vector $\hat{P}$ with coordinates (x, y, z) is calculated as follows [6,8,15,16]:

(8) $$\hat{P}\left(d\right)=\hat{O}+\hat{D}\cdot \left(d+error\left(d\right)+noise\right),$$

where $\hat{O}$ is the ray origin vector; $\hat{D}$ is the direction vector obtained from orientation vectors (Up, Right, Forward) and angles of the sensor; *d* is the intersection distance of the collision; *error* is the equation found in the error model experiment, similar to Equation (7); and *noise* is the random gaussian error *N*(0,3). These data are then organized in the form of synthetic point clouds for each position defined in the Virtual Scene module.

**Figure 5:**
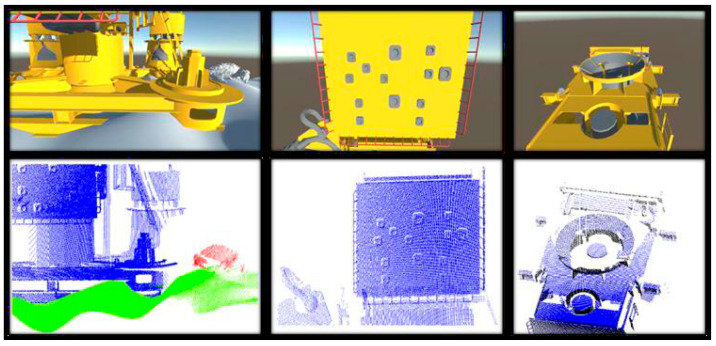
Comparison between virtual scene module camera preview and each synthetic point cloud generated per sensor scanning position [29].

As an example, in the optScan simulator, three camera positions were defined as scanning positions in the Virtual Scene module, as seen in Figure 3. For each of these positions, the module previews the camera device field of view, as shown in the upper boxes in Figure 5. After simulating the virtual scanning inside the Synthetic Point Cloud Generator, each sensor scanning position resulted in a point cloud similar to the elements found in the camera previews. These generated data can have conical distortions, as mentioned in Section 2.3.2.

**Figure 6:**
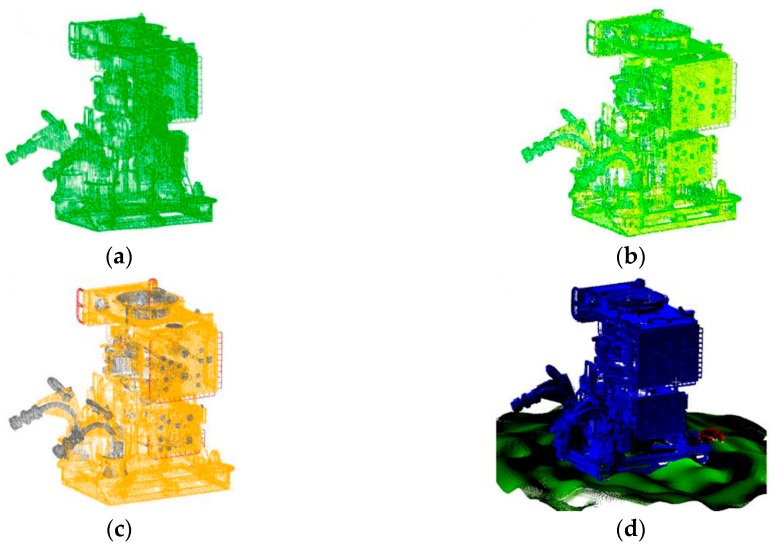
Different properties of the synthetic point cloud: (**a**) 3D points; (**b**) Normal vectors; (**c**) Color; (**d**) ID [29].

Each point in these clouds has the following properties:

- 3D coordinates: Define the position of the point associated with the constructed virtual scene (x, y, z). It is the basic information of a point cloud. The visualization of the points is shown in Figure 6a;
- Normal Vector: These are vectors perpendicular to the surface of the detected objects. Used for material lighting definitions and useful in various deep learning and data segmentation routines [19]. The visualization of the vector in action is shown in Figure 6b;
- Color: Defined in the form of RGB vectors. Useful for checking the point cloud correspondence with the 3D model. Some LiDAR systems already have cameras in the visible spectrum to obtain color information, as the sensor alone cannot acquire these data [10]. The color preview is shown in Figure 6c; and
- Identification Number (ID): This number is a unique identifier defined by the user when creating the scene, as mentioned earlier in this paper. This information facilitates the visualization of different objects within the same cloud, which also facilitates cataloging and data segmentation processes [18,19,20]. The ID preview is shown in Figure 6d.

Given enough scanning positions, it is possible to capture the target of interest in its entirety. However, in most procedures, these one-sided synthetic point clouds need to be unified in a single 3D model [4,6,15,18,30]. Therefore, enters the second step of this module, the Merge application. Here, we suggest developing the application using the Geogram geometric algorithms library [33]. The application receives as input the point clouds selected by the user from those generated in the simulation, then, the data are combined in pairs, and a comparison process between the points existing in these provisional point clouds begins. The purpose of this comparison is to define the existence of redundant points, i.e., points in the dataset with matching 3D position or within a user-defined proximity threshold. This threshold will vary within the range between 0 mm and 1 mm. The efficiency of this method will depend on the metrological parameters configured in the Sensor Behavior module. The redundant points are then grouped and eliminated from the provisional point cloud. This filtering is called space decimation [33].

**Figure 7:**
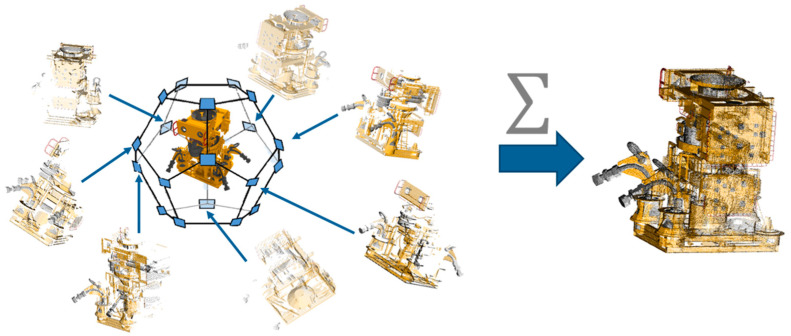
A series of virtual scans are performed, generating a large volume of one-side synthetic point clouds. This dataset is then merged into a combined synthetic point cloud [29].

The process of combining, comparing, and decimating will be repeated until the entire dataset has been transformed into a single point cloud. In the end, the combined point cloud will be output in XYZ and PLY formats. Figure 7 illustrates the process of the Synthetic Point Cloud Generator module.

## 3. Results

### 3.1. Validation

With the sensor simulator developed, it is necessary to validate the quality of the virtual scanning and synthetic point cloud generated. As mentioned earlier, there are two main ways to validate the robustness of the synthetic point cloud generation: comparison of the primary signal of the sensor simulator with the real sensor as a reference; or calculating the error between real and synthetic clouds points positions [4,6,15,18,30]. Here, the first method was chosen, and to better illustrate it, the optScan was used as a case study again.

The process begins with the analysis of the metrological parameters of the chosen reference sensor. In this experiment, the LiDAR [32] was the same used in the development of the error model in the Sensor Simulator module. First, the Sensor Simulator module, with only the measurement model, was configured with the metrological parameters of the Hokuyo LiDAR and a virtual scene based on a simple cylindrical object with low reflectance. Both the on-field and virtual scanning were made with their respective sensors positioned so that the LiDAR central ray hits the center of the object at the same height. A series of scans were carried out, resulting in raw data in the form of intersection distances for both the Hokuyo LiDAR and its digital counterpart.

**Figure 8:**
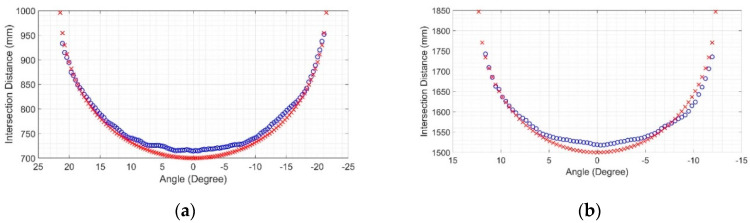
Intersection maps: (**a**) At 700 mm of the sensor’s central ray; (**b**) At 1500 mm of the sensor’s central ray. In both plots, the intersection points of the Hokuyo LiDAR are marked with “o”, while those of the Sensor Simulator (measurement model) are marked with ‘x’ [29].

Later, these raw data were plotted against their rays shooting angles, resulting in intersection maps. As the results were similar in maps from the same LiDAR operation range, only two central ray positions are presented here, one for each LiDAR operation range. Figure 8 shows the intersection map at 700 mm (1st range) and 1500 mm (2nd range), where the intersection points of the Hokuyo LiDAR are marked with ”o”, while those of the Sensor Simulator are marked with “x”. As the distance between sensor and target increases, the number of rays hit decreases, because of the LiDAR laser beam shooting angles.

**Figure 9:**
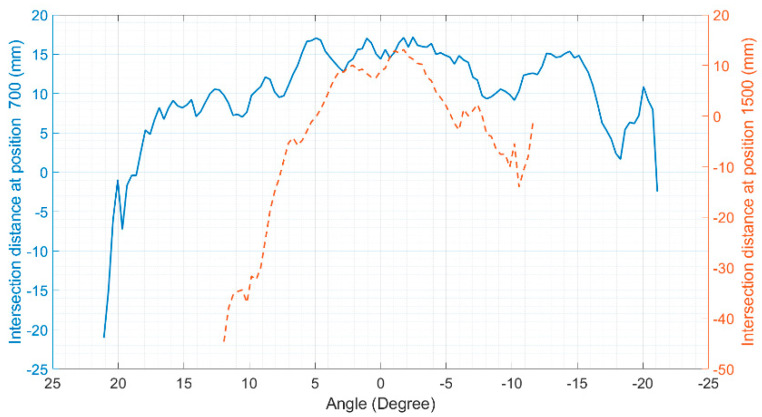
Difference error between real and synthetic data (measurement model only). The continuous line represents errors at 700 mm of the sensor’s central ray. The dashed line represents errors at 1500 mm of the sensor’s central ray.

Once these maps were obtained, the mean squared error between the virtual data and the reference data was calculated for each distance of interest, as shown in Figure 9.

For the position of the sensor central ray at 700 mm, the RMSE found was 11.94 mm, while for the position of the sensor central ray at 1500 mm, the RMSE was 21.83 mm [29]. These calculations only considered the rays that detected the target. These values are good indications of the robustness of the virtual sensor based on measurements since the error between the real and synthetic data is consistent with the measurement uncertainties specified by the manufacturer. This is expected since the simulation had no noise or disturbances, and the environment of the experiment in the real world was controlled to have the least possible impact on the measurements.

**Figure 10:**
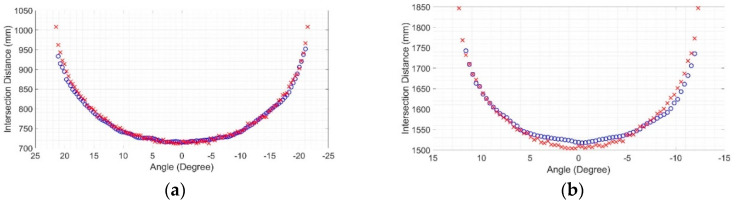
Intersection maps: (**a**) At 700 mm of the sensor’s central ray; (**b**) At 1500 mm of the sensor’s central ray. In both plots, the intersection points of the Hokuyo LiDAR are marked with ‘o’, while those of the Sensor Simulator (hybrid model) are marked with “x” [29].

**Figure 11:**
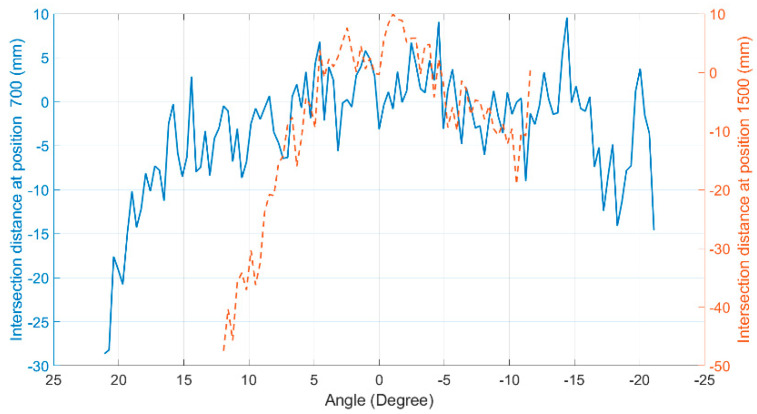
Difference error between real and synthetic data (hybrid model). The continuous line represents errors at 700 mm of the sensor’s central ray. The dashed line represents errors at 1500 mm of the sensor’s central ray.

When the whole hybrid model was assembled, this same validation process was repeated, achieving the following intersection maps in Figure 10, and the mean squared error for each distance of interest was found using the difference between the virtual data and the reference data, shown in Figure 11.

For the position of the sensor central ray at 700 mm, the RMSE found was 7.22 mm, while for the position of the sensor central ray at 1500 mm, the RMSE was 14.13 mm [29]. It can be observed that, when activating the error model in combination with the measurement model, the result of the optScan simulation practically matches the result of the Hokuyo LiDAR scanning.

This proves the robustness of the entire simulator, especially the sensor behavior model, which has shown to behave similarly to a real LiDAR sensor. Besides, this whole process works as a calibration process for the virtual sensor compared to the reference sensor, assuming that it has previously been correctly calibrated [29].

### 3.2. Applications

With the simulator validated, a few applications were tested with two different computer settings [29]:

- Machine A: Intel Core i7–7500U, Nvidia GeForce 940MX, and 16 GB RAM, (Dell/Rio de Janeiro/Brasil).
- Machine B: Intel Core i7–8700K, Nvidia GeForce GTX 1070 Ti 6 GB, and 32 GB, (No brand (self-assembled with parts procured in Rio de Janeiro, Brazil))

The results are shown in the next subsections.

#### 3.2.1. Oil & Gas Subsea Survey

The Oil & Gas industry has been looking for more effective solutions for the underwater inspection carried out in offshore oil fields, due to the hostile conditions of the underwater environment, which affect the quality of the images obtained [12,14,34]. One of these solutions involves using 3D imaging technologies, especially LiDAR devices, for the generation of detailed point clouds.

These datasets are used to evaluate the evolution of the equipment conditions and positions in the oil field. Operations planned with this information significantly reduce costs and risks [12,14,34]. However, the ocean floor is an extremely hostile environment, hindering the massive acquisition of data, consequently preventing the application of AI training techniques that would facilitate the analysis of the structures contained in these point clouds [19]. Virtual scanning can be used to increase the dataset collection.

A simple scene was constructed to represent a small fraction of an offshore oil field with the optScan simulator. The underwater environment optical properties and phenomena were not considered yet, because of the problem complexity. Just recently, harsh environmental conditions started to appear in the literature [26].

Also, it needs to be pointed out that the present simulator has been developed based on a simpler LiDAR sensor [32], as explained in Section 2.3.2. In order to develop a simulator for a LiDAR specifically used in subsea surveys (such as [14]), the steps described in Section 2.3.2 should be repeated for the specific sensor to be modeled. Nevertheless, this section aims at providing an example of the potential of a LiDAR simulator that is developed based on the guidelines presented in this manuscript.

**Figure 12:**
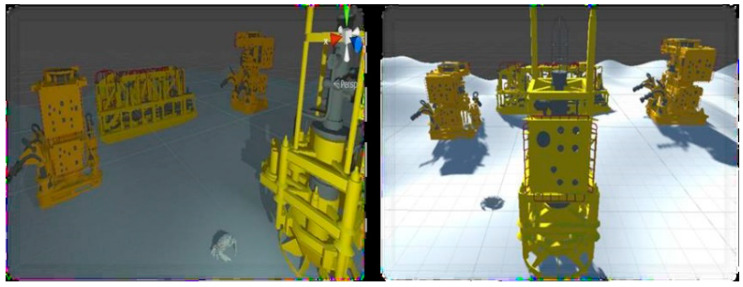
Offshore oil field virtual scene [29].

**Figure 13:**
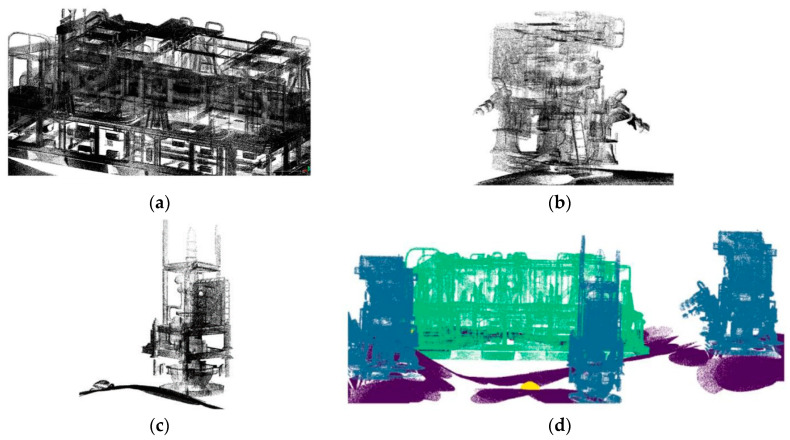
(**a**) Manifold point cloud; (**b**,**c**) Wet Christmas Tree point cloud; (**d**) The merged point clouds with identification property highlighted [29].

The 3D models used were two subsea Christmas tree models, a manifold (equipment used for oil field simplification), a crab (disproportionately enlarged for easy viewing here), and the sandy soil. Within this scene, the sensor’s path was chosen to scan the targets in their entirety. The virtual scanning resulted in a series of point clouds equivalent to the 3D models of subsea equipment. Figure 12 shows the virtual scene, and Figure 13 shows the point clouds generated.

The main objective of generating these synthetic data is to optimize the data acquisition in the field, as the AI trained with this synthetic data will indicate the necessary size of the dataset for training. Thus, the data acquisition can be organized based on the total data required and not on the measurement time [4,18,19].

#### 3.2.2. Semantic Segmentation of Point Clouds

The semantic segmentation technique is the process of classifying each pixel of an image into specific classes. Many technologies are dependent on computer vision and sensing, such as autonomous cars and robotic systems, since these applications need to interpret the environment where they are operating, usually with the use of deep learning [4,15,18,19,20].

**Figure 14:**
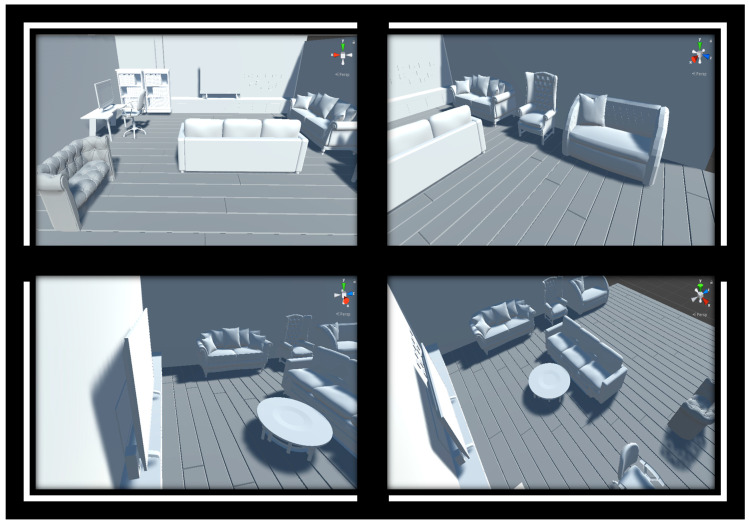
Virtual scene of a living room. The frames display four positions out of a total of eighteen for the virtual sensor [28]. All 3D models were obtained from Free3D [35].

Semantic segmentation can also be applied in the context of point clouds, except that, instead of classifying pixels, it is the points in the dataset that receive class assignments [18,19,20]. However, due to the geometric characteristics of the point clouds, such as the invariance in the permutation of the points in the set and the non-ordering of the elements, the classification process can be complicated and time-consuming [18,19,20]. To facilitate this process, the optScan generates point clouds with a class property assigned to each point. This semantic annotation process is performed indirectly by the user in a semiautomatic approach, passing the class information in the Virtual Scene module, which is annotated at the points detected during the virtual scanning. As an example of this process, a scenario of a room with several objects was built, and several positions were defined for the sensor’s path, as seen in Figure 14.

**Figure 15:**
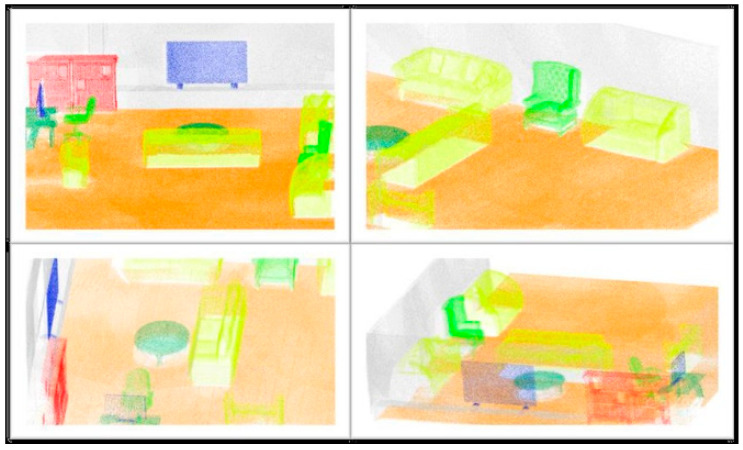
Synthetic point cloud generated with semantic classification seen at different angles. Classes: Red—Bookcase, Yellow—Sofa, Dark green—Table, Light green—Chair, Blue—Monitor/TV, Gray—Wall, and Orange—Floor [29].

The objects were separated into the following seven classes: Floor, Wall, Monitor/Television, Chair, Table, Couch, and Bookcase. The complete simulation process resulted in a complete point cloud of the target scenario, with semantic segmentation information, shown in Figure 15. These synthetic data generated in conjunction with real data assist in the training of deep learning networks for segmentation, increasing the robustness of the process [4,15,18,19,20].

## 4. Conclusions

3D imaging technologies have increasingly widespread use, both in academia and in the industrial sector, especially with the growing computational power, which allows the use of these devices even in the portable form [1,2,3,4,5,6,8,9,10,11,12]. Despite this, the massive demand for imaging data made it necessary to find new forms of acquisition to complement these data, especially in sectors that work with hostile environments or large areas, such as the oil and gas sector in the underwater environment [4,20,34]. Virtual scanning allows meeting this demand for data, reducing cost and production time. However, the focus on the automotive and robotics sectors brought specific solutions to their problems, forcing the development of new simulators whenever a new need is perceived.

The research presented here sought to fill this gap, proposing a modular approach to the simulator, giving its components a certain degree of independence. Additionally, the proposed methods allow the generation of synthetic point clouds with classification data anointed semi-automatically, limited only by the computational power of the equipment where the simulation occurs. In this way, the guideline for sensor simulator development, calibrated by a LiDAR sensor, proved to be effective and robust in the automatic generation of point cloud datasets with classification.

Despite the good results of the guideline, there are some ways to improve it:

- Adding phenomena that the environmental conditions cause to the LiDAR scanning, like light scattering and absorption. This module could work as a filter, changing the properties to match the environment, like a blizzard or underwater turbidity;
- Adding other sources of errors, like those belonging to the construction of the LiDAR device;
- Implementing more LiDAR properties, like beam divergence, beam size, and number of rays per angle, such as described in [28];
- Developing a CCD-based module to capture color information realistically; and
- Using raytracing to include light reflections based on the object’s material.
